# The Impact of Multidisciplinary Discussion (MDD) in the Diagnosis and Management of Fibrotic Interstitial Lung Diseases

**DOI:** 10.1155/2020/9026171

**Published:** 2020-08-15

**Authors:** Ghofran Ageely, Carolina Souza, Kaissa De Boer, Saly Zahra, Marcio Gomes, Nha Voduc

**Affiliations:** ^1^Division of Thoracic Imaging, Department of Medical Imaging, Ottawa Hospital Research Institute, University of Ottawa, 501 Smyth Road, Ottawa K1H 8M2, Canada; ^2^Division of Radiology, Department of Medicine, Rabigh Medical College, King Abdulaziz University, Jeddah, Saudi Arabia; ^3^Division of Respirology, Department of Internal Medicine, Ottawa Hospital Research Institute, University of Ottawa, 501 Smyth Road, Ottawa K1H 8M2, Canada; ^4^Department of Pulmonary and Critical Care Medicine, Stanford University, School of Medicine, 300 Pasteur Drive, Stanford, CA 94305, USA; ^5^Department of Pathology and Laboratory Medicine, Ottawa Hospital Research Institute, University of Ottawa, 501 Smyth Road, Ottawa K1H 8M2, Canada

## Abstract

Accurate diagnosis of interstitial lung disease (ILD) is crucial for management and prognosis but can be challenging even for experienced clinicians. Expert multidisciplinary discussion (MDD) is considered the reference standard for ILD diagnosis; however, there remain concerns regarding lack of validation studies and relative limited information on the impact of MDD in real-life clinical practice. The goal of this study was to assess the effect of MDD in providing a specific ILD diagnosis, changing the diagnosis provided upon referral, and to determine how often and in which way MDD altered management. *Material and Methods*. Retrospective observational study in an ILD referral tertiary academic center. MDD diagnoses were categorized as specific, provisional, and unclassifiable ILD. Pre-MDD and MDD diagnoses were compared for change in diagnosis and concordance rates for specific diagnoses. Relevant change in management including initiation or change in pharmacological treatment, referral to surgical biopsy, and nonpharmacological management were recorded. *Results*. 126 cases were included (79M, 47F, 36–93 years, mean 70 y). Specific MDD diagnosis was provided in 62% (78/126); 12% (15/126) had provisional diagnosis, and 21% (27/126) was unclassifiable. Overall agreement for specific pre-MDD and MDD diagnosis was 41% (52/126) and 80% for idiopathic pulmonary fibrosis (IPF) diagnosis. MDD altered diagnosis in 37% (47/126) and changed management in 39% (50/126). Amongst concordant diagnoses, management was altered in 46% (24/52). In summary, MDD provided a specific diagnosis discordant with pre-MDD diagnosis in a significant proportion of cases and was particularly valuable in the diagnosis of non-IPF ILD. MDD often altered management and had relevant impact on management even in cases with concordant pre-MDD diagnosis.

## 1. Introduction

Interstitial lung diseases (ILD) are a heterogenous group of diseases with varied causes, treatment, and prognoses. Accurate diagnosis of ILD is crucial for management decision and prognosis; however, it can be challenging due to the diverse manifestations and lack of defined diagnostic criteria for some ILDs. Patients with ILD require a dynamic multidisciplinary assessment for integration of clinical, radiological and, at times, pathological findings. The importance of multidisciplinary discussion (MDD) has been emphasized since the publication of the American Thoracic Society (ATS) and European Thoracic Society (ERS) consensus statement on the classification of idiopathic interstitial pneumonias in 2002 [1], and it was endorsed in the updated 2013 ATS/ERS/JRS/ALAT classification [2]. MDD is now considered the reference standard for ILD diagnosis, supplanting histology. Over the last decade, the value of MDD has been documented [3–6], and MDD meetings have been implemented in centers of expertise, becoming the cornerstone of diagnosis and management of ILD. MDD has been shown to improve interobserver diagnostic agreement and lead to change in the final diagnosis [7–9]. Worth noting, however, while the value of MDD seems undeniable, there remain concerns regarding the diagnostic accuracy of MDD and the lack of validation studies. Notably, there is relatively limited information on the clinical impact of MDD in patient care for both final diagnosis and management.

The goal of this study was to determine the impact of MDD in the diagnosis and management of patients with ILD. Specifically, the study assessed the ability of MDD to provide a specific diagnosis, the proportion of cases in which MDD altered the final diagnosis, and the concordance between pre-MDD and MDD diagnoses. The study also assessed how often and in which way MDD altered the management plan.

## 2. Material and Methods

This retrospective observational study was conducted at The Ottawa Hospital, a tertiary academic center with expertise in ILD, and was approved by the institutional ethics review board; informed patient consent was waived. Cases were retrieved from the ILD-MDD database from January 1^st^, 2013, to May 31^st^, 2018. This prospectively maintained database of cases of ILD reviewed in face-to-face weekly/biweekly meetings. The MDD team consists of respirologists, a registered nurse, a thoracic pathologist, and a thoracic radiologist all with expertise in ILD who review clinical information, imaging studies and pathology slides when available, of patients seen at the ILD clinic or referred from the community. Inclusion criteria for the study consisted of patients with suspected or confirmed fibrotic ILD with available recent CT scan of the chest, database entry containing pertinent clinical and pathological information, and documented MDD consensus diagnosis. Patients with confirmed diagnosis of collagen vascular disease, lack of documented consensus diagnosis, or with no CT scan of the chest were excluded.

Information regarding patients' demographics, origin of referral, diagnosis at the time of referral (pre-MDD diagnosis), management before referral if any, consensus diagnosis (MDD diagnosis), and management following MDD were obtained from the ILD-MDD database and, when not available, extracted from patient's electronic records. Origin of referral was documented according to medical specialty and type of practice (non-MDD academic physician or community-based physician). Pre-MDD diagnoses were based on clinical diagnosis provided by referring physician and/or HRCT diagnosis when available. Specific pre-MDD diagnoses were considered valid and recorded when in accordance to the current consensus classification for idiopathic interstitial pneumonias [2]. Patients referred without a specific diagnosis or simply as ILD were classified as ILD-not otherwise specified (ILD-NOS).

MDD consensus diagnosis strictly followed current consensus guidelines for diagnosis and management of IIP (idiopathic interstitial pneumonias) and IPF [2, 10, 11] or current accepted evidence for alternate diagnoses. The terminology applied was in accordance with the diagnostic ontology recently proposed by an International Working Group [12] that categorizes levels of confidence in the diagnosis of fibrotic ILD as confident diagnosis, “provisional” diagnosis, and unclassifiable ILD. Notably, provisional diagnosis included cases with a leading diagnosis (most likely diagnosis) made with acceptable level of confidence sufficient to justify disease-specific therapy. Patients without a specific or provisional diagnosis after first MDD were deemed unclassifiable, and the reason for an unclassifiable diagnosis was recorded when available. Patients with ILD and features of autoimmunity but without established diagnosis of connective tissue disease were classified as IPAF (interstitial pneumonia with autoimmune features) as per current proposed classification.

Pre-MDD diagnoses were compared to MDD consensus diagnoses, change in final diagnosis was recorded in percentage (%), and discordant cases were documented. Relevant change in patient's management following MDD diagnosis was recorded, including initiation or change in pharmacological treatment, termination of medical treatment, referral to surgical lung biopsy, and institution of nonpharmacological management.

## 3. Results

During the study time, 172 cases were reviewed in the ILD-MDD meeting. Patients with confirmed diagnosis of collagen tissue disease (CTD, 19 cases) and long-term ILD patients followed by the MDD respirologist and reviewed for instructive purposes (14 cases) were excluded from the analysis; 13 cases were excluded for missing data. A total of 126 cases were included in the final analysis (79 men, 47 women, 36–93 years, mean 70 years, median 69). Patients were referred to ILD-MDD meetings by physicians of various specialties, most commonly community family physicians and respirologists.  Table 1 summarizes the origin of referral.

### 3.1. Pre-MDD and MDD Diagnoses and Diagnostic Agreement

A specific pre-MDD ILD diagnosis was provided in 59% (74/126) of patients, most commonly IPF (24/74, 32%) and HP (20/74, 27%); the remainder of the patients (52/126, 41%) were referred without a specific diagnosis (ILD-NOS).

When available, the pre-MDD diagnosis was based on a clinical diagnosis made by the referring physician in 45 patients; in the remainder 81 cases, pre-MDD diagnosis was based on initial HRCT diagnosis which in some cases corresponded to the clinical diagnosis provided by the referring physician. A summary of pre-MDD diagnoses is provided in Table 2.

A specific MDD diagnosis was provided in 62% (78/126) of cases; 12% (15/126) had a provisional diagnosis established at initial discussion sufficient to direct management. ILD was deemed unclassifiable at initial discussion in 21% (27/126) patients. In 6 patients, there was no clinical and radiological evidence of ILD after MDD with parenchymal changes attributed to atelectasis or poor technique. A summary of post-MDD diagnoses is provided in Table 2.  Table 3 summarizes cases with a provisional diagnosis and the effect on management.

**Figure 1:**
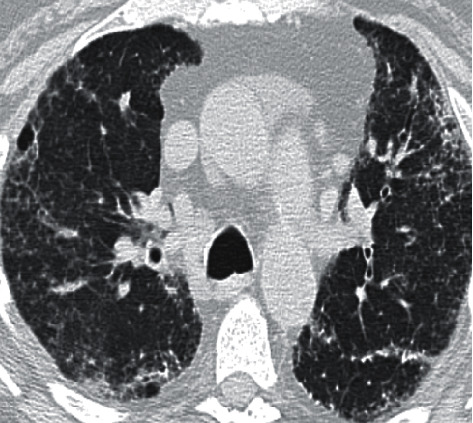
A 72-year old man referred to MDD meeting with a diagnosis of sarcoidosis. HRCT pattern favored chronic HP; however, the case was deemed unclassifiable after multidisciplinary discussion as no exposure was elicited and due to the high clinical probability of IPF. The patient was referred to surgical lung biopsy that demonstrated a usual interstitial pneumonia (UIP) pattern and led to a diagnosis of IPF. The patient was subsequently started on antifibrotic treatment.

The overall agreement for specific pre-MDD and MDD diagnosis was 41% (52/126). MDD altered diagnosis provided upon referral in 37% (47/126) of patients, including 22 specific MDD diagnoses for ILD-NOS. For specific ILD diagnoses, of the 24 patients referred with IPF diagnosis, 20 (80%) were confirmed at initial MDD discussion; one was diagnosed as smoking-related ILD (SR-ILD) and three were unclassifiable (two later diagnosed as UIP/IPF after lung biopsy). Of the 20 patients referred as chronic HP, 12 (60%) were confirmed, including 4 working diagnoses; one patient was diagnosed as SR-ILD, and one was found to have no ILD. Six pre-MDD HP were deemed unclassifiable, and one was later diagnosed as UIP/IPF following lung biopsy (Figure 1).  Table 4 summarizes concordant MDD and pre-MDD diagnoses.

### 3.2. MDD and Effect on Management

MDD led to change in management in 39% (50/126) of patients, notably initiation of medical therapy (30/50), lung biopsy (6/50), and change in medical treatment (4/50), including one patient with no ILD after MDD who had been started on immunosuppression and discontinued after consensus discussion. A total of 21 patients continued medical treatment initiated before MDD (5 with antifibrotic therapy and 16 with immunosuppressant). In the remainder of cases, patients were referred to smoking-cessation program and further rheumatological investigation, and one was referred to lung transplant. Amongst cases with concordant pre-MDD and MDD diagnoses, management was altered in 46% (24/52), either initiation or change in medical treatment. Consensus discussion endorsed pharmacological therapy instituted before referral in 41% (52/126) cases. In 21% (27/126) of patients, MDD recommendation was continued clinical follow up along with referral to smoking-cessation program in eight cases.

Lung biopsy was indicated in 22 (17%) of patients but performed in only 6 cases; 3 patients declined biopsy, and 13 were deemed not eligible to biopsy due to advanced disease. Of the 6 unclassifiable cases with lung biopsy, 4 were diagnosed with UIP/IPF, one with NSIP, and one with UIP pattern and areas of organising pneumonia.

## 4. Discussion

Multidisciplinary discussion established a specific ILD diagnosis in 62% of our patients. When compared to diagnosis upon referral, MDD provided a new diagnosis or altered preexisting diagnosis in 37% of the cases. Our results, corroborating previous studies that showed change in diagnosis following MDD in 40%–53% of cases [6, 8, 13–15], emphasize the importance of expert review and of an integrated radiological and clinical approach in patients with chronic ILD.

A substantial proportion of patients were referred to MDD for evaluation of ILD not specified. For those with a specific pre-MDD diagnosis, concordance rate with consensus MDD diagnosis was 41%. The highest concordance rate was for the diagnosis of IPF (81%), the commonest diagnosis provided by referring physicians. In the seminal study by Flaherty et al. [8], the first to assess the impact of an integrated expert approach in ILD, diagnostic agreement between community and academic physicians was the highest for IPF, with community physicians more likely to provide an IPF diagnosis. In a subsequent multicenter study [3], inter-MDD agreement for diagnosis of IPF was good and only marginally better than diagnosis agreement amongst clinicians acting in isolation. IPF is in the center of the diagnostic pathway of diffuse lung diseases, and evidence-based guidelines for IPF are well-established and widely used even amongst nonexpert physicians explaining the high concordance rates when typical HRCT and clinical features are present. There is evidence to suggest that in such cases, particularly when the clinical probability of IPF is high, expert MDD is unlikely to change diagnosis [3, 10, 16]. In contrast to IPF, the concordance rate for the diagnosis of HP was low in our study. Notably, a relative high proportion of cases referred as HP were deemed unclassifiable ILD. HP can be notoriously difficult to diagnose, and diagnostic agreement is poor even amongst experts, in great part due to lack of consensus diagnostic criteria and evidence-based guidelines [7, 13], although efforts toward improving agreement amongst experts have been explored [17].

Strict adherence to diagnostic criteria maximizes diagnostic certainty but in clinical practice may result in a substantial number of unclassifiable cases, potentially leading to delay in diagnosis and treatment. In current practice, when diagnostic criteria are not fully met for a confident ILD diagnosis, expert assessment may be able to provide a leading diagnosis that is more likely than not based on clinical judgement. For such cases, an International Working Group has recently proposed the term “provisional” diagnosis. In our cohort, 12% of the patients did not fulfill diagnostic criteria and received a provisional diagnosis considered adequate to directed management. Including these cases, the percentage of patients with ILD diagnoses post-MDD raised from 62% to 73%.

Cases that cannot be given a confident or provisional diagnosis even after comprehensive MDD are considered unclassifiable ILD [12]. The concept of unclassifiable ILD was endorsed as a formal entity in the 2013 updated classification [2] with reported rates varying from 10% to 44% [13, 15, 18–20], and the large variability was explained by inconsistent definitions and differences in study design [21]. In our cohort, ILD was deemed unclassifiable in 21% of cases, in a considerable proportion due to lack of pathological diagnosis at initial assessment. Of note, some cases categorized as unclassifiable on first MDD review had subsequent biopsy and specific diagnoses. In the study of 938 patients reported by De Saeleer et al. [13], most patients received a definite diagnosis after the second MDD. Thus, selected cases might be better considered as “not yet classified” or “unclassified” rather than unclassifiable ILD, as a definite diagnosis may be achieved once more information is made available, highlighting the importance of a dynamic and iterative approach combined with longitudinal assessment of ILD patients. Worth noting, in a substantial number of patients, a specific guideline-based diagnosis may never be achieved particularly if lung biopsy is contraindicated due to advanced disease, situation encountered in 13 out of 22 (59%) biopsy indications in our cohort. This emphasizes the importance of early referral to centers of expertise in ILD, particularly for patients with initial non-IPF diagnosis who may require additional diagnostic tests and lung biopsy.

MDD altered management in 39% of our patients, mostly initiation of medical therapy. In 6 cases, MDD led to change or termination of medical treatment deemed inappropriate, including one patient receiving immunosuppressant with no clinical or radiological evidence of ILD upon MDD. While comprising a relatively small proportion of patients, the implication of this in an individual case cannot be underestimated. It often affected management even when diagnosis remained the same and for cases deemed unclassifiable after initial MDD. Interestingly, even for cases with concordant pre-MDD and MDD diagnoses, management was altered in a considerable proportion (46%) of patients emphasizing the central role of expert opinion not only for diagnosis but for institution of appropriate treatment, remarkably antifibrotic drugs for IPF and immunosuppressive/anti-inflammatory therapy for non-IPF ILD. Change or initiation of nonpharmacologic management was common and included referral to smoking-cessation programs and supportive treatment (supplemental oxygen and pulmonary rehabilitation).

This study has several limitations, notably the inherent limitations of a retrospective single-center design, including selection and referral bias. The study included only one tertiary center; thus, MDD diagnosis was not validated and not assessed for inter-MDD agreement. Due to the nature of MDD diagnosis, while our study assessed changes in diagnosis, it cannot assess the accuracy of MDD diagnosis. Another limitation is the relative small number of patients with surgical biopsy which otherwise might have increased the rate of specific diagnoses. Finally, there was no longitudinal follow up to evaluate disease behavior and change in clinical or radiological patterns that might have altered diagnosis and management. Thus, the effect of MDD in outcome was not assessed. Nonetheless, we believe our study strengthens the importance of MDD in patient diagnosis and management decisions with key real-life implications including biopsy referral, initiation, and change of therapies.

In summary, MDD provided a specific diagnosis discordant with pre-MDD diagnosis in a significant proportion of cases and was particularly valuable in the diagnosis of non-IPF ILD. MDD altered management in the majority of patients and had relevant impact on management even in cases with concordant pre-MDD diagnosis.

## Figures and Tables

**Table 1:** Origin of referral.

Specialty	Number of cases
General practice	64
Respirology	34
Internal medicine	6
Cardiology	5
Emergency	3
Surgery	3
Intensive care unit	1
Rheumatology	1
Oncology	1
Not specified	8

**Table 2:** Pre-MDD and MDD consensus diagnoses.

Diagnosis	Pre-MDD, *n* (%)	MDD, *n* (%)
ILD-NOS	52 (41%)	0 (0%)
HP	20 (16%)	21 (16.6%)^*∗*^
IPF	24 (19%)	34 (27.0%)^*∗*^
SR-ILD	5 (4.0%)	10 (8.0%)
NSIP	6 (4.7%)	6 (4.7%)^*∗*^
OP	3 (2.4%)	2 (1.6%)
Drug toxicity	4 (3.2%)	4 (3.2%)
Asbestosis	1 (0.8%)	1 (0.8%)
Sarcoidosis	4 (3.2%)	1 (0.8%)
LIP	1 (0.8%)	1 (0.8%)
GL-ILD	1 (0.8%)	1 (0.8%)
Pulmonary ossification	1 (0.8%)	1 (0.8%)
Aspiration	1 (0.8%)	1 (0.8%)
HP/IPF	2 (1.6%)	0 (0%)
DIPNECH	0 (0%)	1 (0.8%)
ILA	0 (0%)	4 (3.2%)
Cystic disease	1 (0.8%)	0 (0%)
IPAF	0 (0%)	5 (4.0%)
Unclassifiable ILD	0 (0%)	27 (21.4%)
No ILD	0 (0%)	6 (4.7%)

^*∗*^Includes provisional diagnoses (HP = 8, IPF = 4, and NSIP = 3). ILD = interstitial lung disease; ILD-NOS = interstitial lung disease-not otherwise specified; HP = hypersensitivity pneumonitis; IPF = idiopathic pulmonary fibrosis; SR-ILD = smoking-related ILD; NSIP = nonspecific interstitial pneumonia; OP = organising pneumonia; LIP = lymphocytic interstitial pneumonia; GL-ILD = granulomatous–lymphocytic interstitial lung disease; DIPNECH = diffuse idiopathic pulmonary neuroendocrine cell hyperplasia; ILA = interstitial lung abnormality; IPAF = interstitial pneumonia with autoimmune features.

**Table 3:** Provisional diagnoses and effect on management.

Provisional diagnosis (*n*)	Effect on management (*n*)
HP = 8	Initiation pharmacological treatment (4)
	No change (4)

IPF = 4	No change (4)

NSIP = 3	No change (2)
	Initiation pharmacological treatment (1)

HP = hypersensitivity pneumonitis; IPF = idiopathic pulmonary fibrosis; NSIP = nonspecific interstitial pneumonia.

**Table 4:** Concordant Pre-MDD and MDD diagnoses.

Concordant diagnoses	*N*	%
HP	12	23
IPF	19	37
SR-ILD	4	7
NSIP	5	9
OP	2	4
Drug toxicity	3	6
Asbestosis	1	2
Sarcoidosis	2	4
LIP	1	2
GL-ILD	1	2
Pulmonary ossification	1	2
Aspiration	1	2

^*∗*^Includes provisional diagnoses (HP = 4, NSIP = 1). HP = hypersensitivity pneumonitis; IPF = idiopathic pulmonary fibrosis; SR-ILD = smoking-related ILD; NSIP = nonspecific interstitial pneumonia; OP = organising pneumonia; LIP = lymphocytic interstitial pneumonia; and GL-ILD = granulomatous–lymphocytic interstitial lung disease.

## Data Availability

The data used to support the findings of this study are restricted by The Ottawa Hospital Research Ethics Board in order to protect patients' privacy. Data are available from the corresponding author for researchers who meet the criteria for access to confidential data.
